# Artificial Bee Colony algorithm in estimating kinetic parameters for yeast fermentation pathway

**DOI:** 10.1515/jib-2022-0051

**Published:** 2023-06-22

**Authors:** Ahmad Muhaimin Ismail, Muhammad Akmal Remli, Yee Wen Choon, Nurul Athirah Nasarudin, Nor-Syahidatul N. Ismail, Mohd Arfian Ismail, Mohd Saberi Mohamad

**Affiliations:** Artificial Intelligence and Bioinformatics Research Group, Faculty of Computing, Universiti Teknologi Malaysia, 81310 Skudai, Johor, Malaysia; Institute for Artificial Intelligence and Big Data, Universiti Malaysia Kelantan, 16100 Kota Bharu, Kelantan, Malaysia; Faculty of Data Science and Computing, Universiti Malaysia Kelantan, 16100 Kota Bharu, Kelantan, Malaysia; Health Data Science Lab, Department of Genetics and Genomics, College of Medical and Health Sciences, United Arab Emirates University, P.O. Box 15551, Al Ain, United Arab Emirates; Faculty of Computing, College of Computing & Applied Sciences, Universiti Malaysia Pahang, 26300 Gambang, Pahang, Malaysia

**Keywords:** artificial bee colony algorithm, artificial intelligence, bioinformatics, data science, fermentation pathway, parameter estimation

## Abstract

Analyzing metabolic pathways in systems biology requires accurate kinetic parameters that represent the simulated *in vivo* processes. Simulation of the fermentation pathway in the *Saccharomyces cerevisiae* kinetic model help saves much time in the optimization process. Fitting the simulated model into the experimental data is categorized under the parameter estimation problem. Parameter estimation is conducted to obtain the optimal values for parameters related to the fermentation process. This step is essential because insufficient identification of model parameters can cause erroneous conclusions. The kinetic parameters cannot be measured directly. Therefore, they must be estimated from the experimental data either *in vitro* or *in vivo*. Parameter estimation is a challenging task in the biological process due to the complexity and nonlinearity of the model. Therefore, we propose the Artificial Bee Colony algorithm (ABC) to estimate the parameters in the fermentation pathway of *S. cerevisiae* to obtain more accurate values. A metabolite with a total of six parameters is involved in this article. The experimental results show that ABC outperforms other estimation algorithms and gives more accurate kinetic parameter values for the simulated model. Most of the estimated kinetic parameter values obtained from the proposed algorithm are the closest to the experimental data.

## 1 Introduction

One of the main goals of systems biology is to develop a model that can act as a biological function simulator. It is advantage to determine whether combining individual metabolite properties can give better explanations of the fermentation pathway without conducting a real experiment [1, 2]. Hence, it is crucial to increase the competency of the simulation process. Nonlinear differential equations are highly complex, so parameter estimation is difficult with the increasing size and nonlinearity of the fermentation pathway, leading to a corresponding increase in size and variable in the model [3]. Furthermore, the complicated equation increases the complexity of the model [4]. Parameter estimation is one of the critical steps in constructing a mathematical model. Unfortunately, it possesses several problems; (1) the existence of noisy data leads to low accuracy, and (2) the increasing number of unidentified parameters and equations in the model makes it a complex model.

The field of systems biology has seen rapid development in recent years, and various mathematical models have been proposed to simulate biological functions, including the fermentation pathway in *Saccharomyces cerevisiae*. Parameter estimation is a critical step in constructing such mathematical models, and it is essential to obtain accurate parameter values to ensure the accuracy of the simulation results. Several algorithms have been proposed in the literature for parameter estimation in biochemical systems, including Simulated Annealing, Simplex algorithm, and Scatter Search. However, these algorithms need to be revised to handle the complexity and nonlinearity of the models, leading to inaccurate parameter estimates. To address this issue, researchers have explored the use of metaheuristic algorithms, such as Particle Swarm Optimization, Genetic Algorithm, and Ant Colony Optimization. These algorithms have shown promising results in various optimization problems, including parameter estimation in biochemical systems. However, there is still room for improvement in terms of their efficiency and effectiveness.

Previously, algorithms such as Simulated Annealing (SA), Simplex algorithm, and Scatter Search [5, 6] were used for parameter estimation. Parameter estimation using the SA algorithm was applied in the S-system model of a biochemical network proposed by Gonzalez et al. [7]. In contrast, Zhou et al. [8] introduced the Simplex algorithm in parameter estimation for intracellular uptake and delivery of plasmid DNA. Remli et al. [5, 6] proposed a Scatter Search to solve large-scale parameter estimation in kinetic models of biochemical systems. Nevertheless, those algorithms still face some problems. Simplex algorithm and SA both require a sufficient number of iterations to guarantee a high-quality solution, and SA is also sensitive to parameter selection [9, 10]. Scatter Search needs help to satisfy many nonlinear constraints to high accuracy [11]. Hence, the accuracy of the simulated parameter value may decrease due to the incapability of the algorithms to find a better solution in the search space.

While existing algorithms have shown promising results in parameter estimation for biochemical systems, they have limitations in handling the complexity and nonlinearity of the models. Simulated Annealing and Simplex algorithm require a large number of iterations to guarantee a high-quality solution, and Scatter Search faces difficulty in satisfying many nonlinear constraints to high accuracy. Additionally, Particle Swarm Optimization, Genetic Algorithm, and Ant Colony Optimization, while showing promise in various optimization problems, have limitations in terms of their efficiency and effectiveness in parameter estimation for biochemical systems. Therefore, there is a need for more efficient and effective algorithms to improve the accuracy of the parameter estimates for the simulated models. In this paper, we propose the use of the Artificial Bee Colony algorithm, which has several advantages, such as global search capability, fast convergence rate, and robustness against noise in the data. The proposed algorithm overcomes the limitations of existing algorithms and shows superior performance in obtaining more accurate kinetic parameter values for the simulated model compared to other existing algorithms.

This paper aims to improve the accuracy of kinetic parameters for the yeast fermentation pathway of *S. cerevisiae*. The existing algorithms mentioned need to be revised to handle the complexity and nonlinearity of the model. Therefore, we chose to explore the potential of the Artificial Bee Colony algorithm, which is inspired by the foraging behavior of honeybees. This algorithm effectively solves various optimization problems, including parameter estimation in the kinetic model of biochemical systems. The ABC algorithm has the advantage of being able to explore the search space more efficiently and effectively. In this paper, we compare the performance of the ABC algorithm with other existing algorithms and demonstrate its superiority in obtaining more accurate kinetic parameter values for the simulated model.

In Section 2, the methodology is briefly explained, whereas, in Section 3, the experimental setup is described. In Section 4, the result and discussion of this study are presented. Lastly, in Section 5, the conclusion of this work is drawn, and future works are suggested.

## 2 Methodology

### 2.1 Problem formulation

In the case of a biochemical process, hypotheses based on the knowledge of the underlying network structure of a pathway are translated into a system of kinetic equations, and parameters are obtained from literature or estimated from a data fit. Parameter estimation can be formulated as Equation (2.1): in the model noted as s(X) and a bio-logical compound shown as s has a set of parameters which is *X* = {*X1,X2,X3, …, XD*} where *D* is the total number of parameters. Hence the formula for reaction rate of compound s can be formulated as below:

$$\frac{ds}{dt}=g\left(s\left(X\right),t\right),$$

$$s\left(t_{0}\right)=s\left(0\right),$$

(2.1) $$y=g\left(s\left(X\right),t\right)+e$$

In Equation (2.1), g represents a nonlinear function that is involved in forming an ordinary differential equation (ODE), and t represents sampling time. Then, y is a time series of simulated data, also known as the model’s output, and e represents the noise data randomly produced by Gaussian noise *N* (1,0). The purpose of parameter estimation is to find the set of the optimal parameter, which can be noted as *X*. Then variance between simulated time-series data stated as y and experimental time-series data noted as *y* ^exp^ can be reduced. The variance can be calculated by applying the nonlinear least squared error function, *f*(*X*) which is shown below:

(2.2) $$f\left(X\right)=\min\overset{n}{\underset{i=1}{\sum }}{\left(y^{\exp}-y\right)}^{2}$$

Equation (2.2), *n* is the maximum value generated, and *i* is the index variable.

### 2.2 Artificial Bee Colony algorithm

In 2005, the first ABC was proposed by Karaboga [12]. The algorithm is based on mimicking the foraging honeybee behavior. Three components inside ABC comprise the food source, employed bee, and unemployed bee. In ABC, a solution is represented by a food source, whereas the bee represents the agent in finding a solution. The employed bee goes to a food source that has been visited previously, whereas the unemployed bees wait and decide to choose a food source, and the scout bees search for a solution randomly.

**Figure 1:**
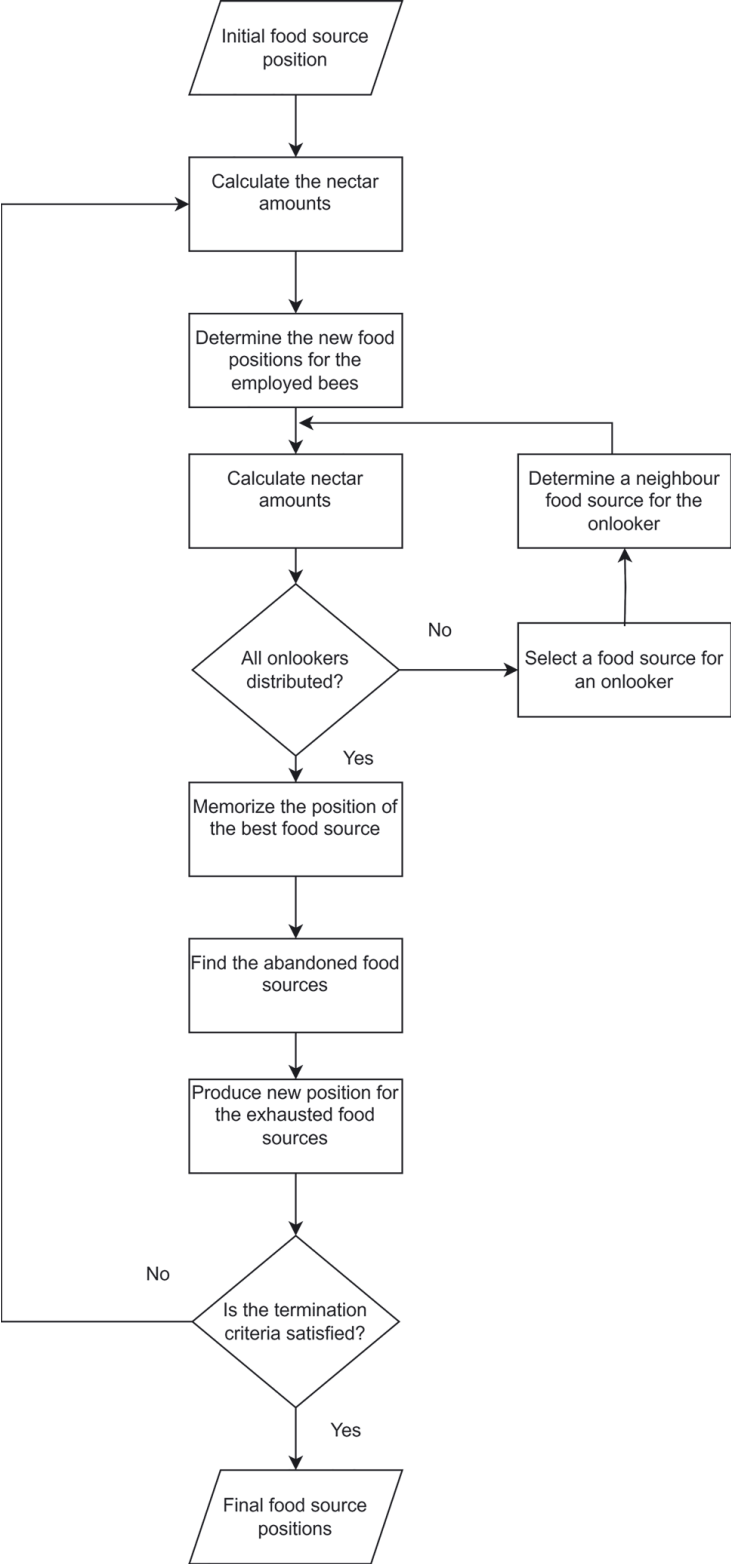
ABC flowchart.

The fitness of the solution is represented by the amount of nectar inside the food source. The number of employed bees in a population is the same as the number of solutions. Figure 1 shows the flowchart of ABC.

#### 2.2.1 Initialization phase

Firstly, all variable values are initialized based on problem, which are *i* = number of kinetic parameters to be optimized, *j* = 10, *xi*Max = initial guess*10, *xi*Min = initial guess/10, Maxtrial = 10, and Maxcycle = 20. The first step is the initialization of the population with the matrix of *i × j*, where *i* is denoted as the number of reactions in the kinetic model, and *j* is denoted as the number of possible solution populations. The number of *j* must be more than *i* because *j* equals the number of employed bees.

(2.3) $$x_{ii}=x_{i}+rand\left(0,1\right)\left(x_{i}\min-x_{i}max\right)$$

Equation (2.3) is the initial food source (*x*_*ii*_) where *x*_*i*_ is the minimum value *x*_*i*_ max is the maximum values of parameter boundaries (lower and upper bounds). Then, the initial food source is created. The population is then evaluated to get the fitness value of each possible solution using a fitness function.

#### 2.2.2 Employed phase

In this phase, a new population is created randomly near the original position populations. The amount of new candidate population is the same as the amount in the first phase. The formula ABC of the new neighboring food source (*v*_*ii*_) is defined by Equation (2.4):

(2.4) $$v_{ii}=x_{ii}{+\emptyset }_{ii}\left(x_{i}+x_{ki}\right)$$

Where the initial population is noted by *x*_*ii*_, and ∅_*ii*_ is a uniformly distributed real random number in the range [1,−1] and k is a random number between range 1 to size population that the index chosen has to be different. The formula for fitness (fitness_*i*_) calculation is shown as Equation (2.5):

$$\mathrm{fi}tness_{i}=1\left(\frac{1}{1-f_{i}}\right)if\;f_{i}\geq 0;$$

(2.5) $$\mathrm{fi}tness_{i}=1+abs\left(f_{i}\right)if\;f_{i}<0;$$

where *f*_*i*_ is the value of the food source obtained. The new population’s fitness is also evaluated using the given fitness formula. Both populations, the new candidate and the present population fitness, are compared based on the local selection process, also known as the greedy selection process. Employed bees carry out this selection process to select the best fitness between the two populations. If the new one is better than the present one, the present one will be forgotten, and the new candidate will be memorized, and vice versa.

In this phase, the number of cycles is a control parameter. A trial is predefined for each solution. The initial or the original populations might have higher fitness values compared to the newly formed solution population. Usually, this happens during the exploitation of the solution population. In this situation, the current solution cannot be improved, and the control parameter is used to limit the abandonment. The trial increases by one if the current solution cannot be improved, or the trial will be zero in the vice versa situation. The current solution that cannot be further improved beyond the trial limit is considered exhausted and abandoned by the employee bees.

#### 2.2.3 Onlooker phase

A new population is generated in this phase from the possible solutions where the employee phase has developed. The formula for probability (*p*_*i*_) used is shown in Equation (2.6):

(2.6) $$p_{i}=\mathrm{fi}t_{i}/\overset{\text{SN}}{\underset{i=1}{\sum }}\mathrm{fi}t_{i}$$

Whereby SN is denoted as population size, fit_*i*_ denoted as fitness value in i iteration, and fit_*i*_ is denoted as fitness value in *j* iteration. The number of onlookers must be the same size as the number of employee bees, so the number of the new population is the same as the previous one. Based on the given formula, the possible solution can be estimated using the probability of the population solution weighted. If a higher probability is achieved, there is a higher chance that the solution can be achieved. When the present population is of higher fitness value, it will replace the old one or vice versa.

Selecting a population from two populations using the greedy selection process is essential. Hence, the population with a higher fitness value replaces the old one. The value of *p* needs to be recalculated to decide whether the present population needs to be replaced or remain. This process is iterated to get the best population among the two populations. It is like the employee phase, and the control parameter is used to limit the abandons of the population. If the population cannot be improved, the trial increases by one, whereas a replaced population trial is set to zero.

#### 2.2.4 Memorize the best phase

The best fitness value among the populations is selected by using the greedy selection algorithm. The artificial bee memorizes the position and shares her information with onlooker bees, and the position of the food source is updated, otherwise is kept the same. The selected population represents the optimal value for the parameter.

#### 2.2.5 Scout phase

Every food source has only one employed bee; therefore, the number of employed bees is equal to the number of food sources. If the position cannot be improved through the predetermined number of trials “limit” of bees, the food source is abandoned, and its employed bee becomes a scout. The scout starts to search for a new food source randomly, and after finding a new source, its new position is accepted. The algorithm terminates if it reaches the maximum cycle or it meets the termination condition.

## 3 Experimental setup

This paper obtains a kinetic model of the *S. cerevisiae* fermentation pathway from the BioModels database [13] in SBML format. This model has several components, such as species, reaction, and global parameters.

This model has an extracellular and cytoplasmic compartment type. These two compartments are three-dimensional compartments with a constant of one liter size. The properties are set to true to prevent the amount from being affected by any reaction. Table 1 shows the properties of each species. The experimental and estimated kinetic parameter values generated are used in the ODE of glucose shown in Equation (3.1).

(3.1) $$\frac{d}{dt\left(Glci\right)}=\frac{+Vin-Vhk}{cytoplasm}$$

where Glucose inside the cell is represented by Vin, which has Vm1 and KilG6p as parameter properties. Vhk is a hexokinase involved in the production of glucose and ATP by conversion of glucose-6-phosphate in a reversible reaction. These two reactions are shown below.

$$Vin=cytoplasm*\left(Vin_{\text{Vml}}-Vin_{\text{KilG}6\text{P}}*G6P\right)$$

$$\begin{array}{ll} Vhk & =cytoplasm*Vhk\text{\_}Vm2/\left(1+Vhk\text{\_}Km2Glc/Glci+Vhk\text{\_}Km2ATP/ATP\right.\\ & \;\left.+Vhk\text{\_}Ks2Glc*Vhk\text{\_}Km2ATP/\left(Glci*ATP\right)\right) \end{array}$$

**Table 1:** Properties for each species.

Id	Name	Compartment	Derived unit
Gly	Glycerol	Cytoplasm	mmol/L
EtOH	Ethanol	Cytoplasm	mmol/L
Carbo	Glycogen and trehalose	Cytoplasm	mmol/L
Glco	Clucose outside the cell	Extracellular	mmol/L

The performance evaluation of ABC, SA, and Simplex algorithms in estimating parameter values are evaluated using standard error rate (A) and standard deviation (STD) for 50 runs. The algorithms were executed in MATLAB R2010a on a 2048 MB RAM and Intel Pentium 4 processor laptop. For ABC, the colony size was set to 40, the limit of the total number of trials for each employed bee was set to 30, the onlooker bee was set to 50, and the bee was set to 70. The number of function evaluations is equal to the number of runs. This is because the function is evaluated once per run. For 50 runs, 50 functions are evaluated.

The time series for glucose is obtained from Equation (3.1). The formula of evaluation performance is given as Equation (3.2).

Error rate *e*:

(3.2) $$e=\overset{N}{\underset{i=1}{\sum }}{\left(y-yi\right)}^{2}$$

Where *N* is the number of runs whereas y and yi are experimental and simulated parameter values respectively.

Average error rate (*A*):

(3.3) A=e/N

Where *e* and *N* represent the error rate and the number of runs. The simulated parameter value is close to the experimental value if the average error rate is small.

Standard deviation (STD):

(3.4) $$\text{STD}=\sqrt{e/N}$$

Where *e* is the error rate, and *N* is the number of runs. An accurate simulated parameter value is obtained if the STD value is closer to zero.

## 4 Results and discussion

This paper presents a comparison between ABC, the Simplex algorithm, and SA in estimating parameter values. Simplex algorithm and SA are chosen because both algorithms are widely used in parameter estimation problems in biological models, engineering, and hydrogeology. Accuracy evaluation is done by generating time series data for glucose inside the cell. The calculation of the average error rate and the standard deviation is carried out by using the time series data. High standard deviations indicate low precision and low standard deviations indicate high precision. The production graph, with a comparison of three estimation algorithms, is produced.

The simulated result values are used to calculate glucose’s average error rate and standard deviation. Table 2 shows the result for glucose and the value of average error rate (*e*) and STD.

**Table 2:** Average of error rate and STD values for glucose.

Evaluation criteria	ABC	Simplex	SA
Average of error rate, *A*	7.84 × 10^−6^	9.7344 × 10^−4^	7.182 × 10^−4^
STD	0.0028	0.0312	0.0268

The bold numbers represent the best result.

Table 2 shows that ABC has the lowest average error rate and the lowest STD value for glucose, with a value of 7.84 × 10^−06^ and 0.0028, respectively, whereas SA has an average error rate of 7.1824 × 10^−04^ and a STD value of 0.0268. For Simplex algorithm, it has an average error rate of 9.7344 × 10^−04^ and an STD value of 0.0312, that is the highest among the algorithms. The standard deviation measurement shows how different the value is dispersed from the mean average value. The STD value of ABC shows that it is closer to the average value, indicating that the simulated result produced by ABC is consistent over time.

**Figure 2:**
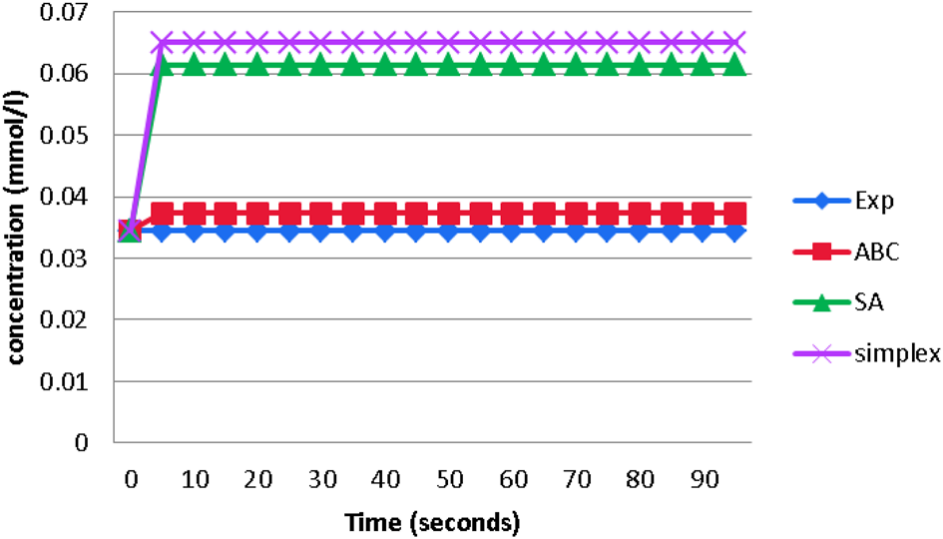
Concentration glucose against time in second.

The time-series data for concentration glucose is generated and shown in Figure 2. The figures comprise the experimental data and the simulated results obtained by ABC, Simplex algorithm, and SA.

In Figure 2, the *x*-axis represents the time, and the *y*-axis represents concentration. Based on Figure 2, the line for ABC is the closest to the experimental line, followed by the SA and Simplex algorithm line. The graph shows that ABC is the most accurate compared with the other two algorithms. In contrast, the Simplex algorithm and SA display a distance from the experimental line.

The outcome of the result proves that the estimated values of the kinetic parameters from ABC successfully enhance the production rate since the line is slightly higher compared to the experimental. The result means there is an enhancement in the concentration for both metabolites compared to the other two previous works.

ABC shows a better performance in enhancing glucose production as it is good in finding optimum parameter values. ABC can avoid being trapped in local optima due to its ability to find new solutions if the current solution cannot be improved (exceeding the maximum trial). Thus, the results show that ABC is more accurate and reliable than SA and Simplex algorithms. From the results, SA shows poor performance in finding the optimal value of the parameters in the fermentation pathway model compared to ABC. Rutenbar [14] found that the SA algorithm could be faster due to its iterative improvement in nature. Many configurations are required to be visited at many temperatures to achieve the optimal solution. Moreover, the SA algorithm can only find the global minimum if the movements are sufficient, but there is a risk that it might not converge in real problems. The Simplex algorithm shows the poorest performance compared to ABC and SA algorithms. The Simplex algorithm does not depend on the gradient, which means converging slowly or not converging at all is possible [15].

## 5 Conclusion and future work

In this paper, we propose the use of the Artificial Bee Colony algorithm, which has several advantages, such as global search capability, fast convergence rate, and robustness against noise in the data. The proposed algorithm overcomes the limitations of existing algorithms and shows superior performance in obtaining more accurate kinetic parameter values for the simulated model compared to other existing algorithms.

There is still room for improvement in finding the optimum value of parameter values for better simulation and greater accuracy. In conclusion, experimental parameter estimation is time-consuming, complicated, and expensive. However, implementing ABC reduces the computational time for parameter estimation successfully. For further improvement, a new strategy should be adopted for ABC due to its good exploration ability but poor exploitation performance [16]. Furthermore, the variety of evaluation methods can also help determine the algorithm’s performance and have more functions in handling the noise data in the algorithms efficiently [3]. Also, one of the most challenging bottlenecks in whole-cell modeling is parameter estimation because a complete whole-cell model should simulate the functional role of every gene and gene-product. It leads to large-scale metabolic parameter estimations [17]. ABC should be further expanded to that scale to resolve the problem.
